# Impact of Chinese national centralized volume-based drug procurement policy on health costs and utilization: An interrupted time series analysis using panel data (2009–2022)

**DOI:** 10.1371/journal.pone.0338409

**Published:** 2025-12-26

**Authors:** Yishi Jiang, Guangsheng Wan, Yufeng Shi, Guiping Pu, Xiaojun Shao

**Affiliations:** 1 NHC Key Lab of Reproduction Regulation, Shanghai Engineering Research Center of Reproductive Health Drug and Devices, Shanghai Institute for Biomedical and Pharmaceutical Technologies, Shanghai, China; 2 College of Nursing and Health Management, Shanghai University of Medicine and Health Sciences, Shanghai, China; University of Nigeria, Enugu Campus, NIGERIA

## Abstract

**Introduction:**

Chinese volume-based procurement (VBP) policy, implemented nationally in 2019, represents a cornerstone of healthcare reform aiming to reduce drug prices through centralized drug procurement. While existing studies have demonstrated the success of the VBP policy in lowering drug costs, comprehensive assessments of system-wide impacts on healthcare expenditures and service utilization remain underexplored. This study evaluates its effects on outpatient and inpatient service costs and volumes.

**Materials and methods:**

We conducted an interrupted time series analysis using panel data (2009–2022) from the *China Health Statistical Yearbook* and the *China Statistical Yearbook*. Primary outcomes included CPI(Consumer Price Index)-adjusted per-visit outpatient(OP)/inpatient(IP) expenditures, hospital OP rates, hospital IP rates, and length of stay. Segmented regression models quantified immediate and long-term policy effects, with two-stage meta-analysis evaluating regional heterogeneity.

**Results and discussion:**

When the VBP implementation occurred, per-visit outpatient costs (β_2_ = 21.400, P < 0.001) and inpatient costs (β_2_ = 693.749, P = 0.006) showed an instantaneous increasing trend. The post-policy long-term trend was decreasing annually by 5.702 Chinese Yuan (CNY) (P < 0.001) for per-visit outpatient costs and 270.670 CNY (P = 0.012) for per-visit inpatient costs compared to the pre-policy period. Hospital OP rates immediately decreased by 0.510 visits (P < 0.001) while hospital IP rates dropped by 3.775 percentage points (P < 0.001), with length of stay increasing by 0.522 days (P < 0.001) immediately after the policy was implemented. Hospital OP rates had a modest increasing trend (β_3_ = 0.069, P = 0.046). Marked regional heterogeneity was observed (I² = 64.6–88.3%), with municipalities showing the most pronounced variations.

**Conclusions:**

Chinese national centralized volume-based drug procurement policy has significantly reduced drug prices but initially increased non-drug costs. Regional disparities linked to aging demographics and complementary reforms (e.g., Diagnosis Related Group) shaped outcomes, while COVID-19 temporarily suppressed utilization. Future policy optimization should integrate Diagnosis Related Group payment reforms with regionally tailored strategies to balance cost containment and service quality. We recommend enhancing temporal resolution and extending observation periods to enable more precise policy evaluation.

## Introduction

In China, pharmaceutical expenditures constitute a substantial proportion of total health expenditures, accounting for 26.90% in 2023 [1], representing a major source of disease burden. Drug pricing directly affects medication accessibility and population health outcomes. To reduce drug prices and eliminate procurement-related corruption, China initiated centralized drug purchasing in 1993, with progressively increasing centralization levels in recent years [2,3]. Consequently, the share of pharmaceutical expenditures in total health expenditures has shown a consistent decline [1]. In 2018, a pilot Volume-Based Procurement (VBP) program was launched across 11 cities and then all provincial entities engaged in the VBP one year later. As a cornerstone of healthcare reform, the VBP policy aims to lower drug prices through volume-price tradeoffs, eliminate gray costs in circulation, optimize procurement mechanisms, and improve healthcare resource allocation. To date, ten national VBP batches have been implemented, covering 435 drugs with average price reductions exceeding 50%, substantially alleviating patients’ financial burdens [4].

Existing evaluations have primarily focused on the policy’s impacts on drug pricing and medication costs, as well as medical institutions and drug utilization [5]. Most findings confirm that VBP significantly lowered prices and improved affordability [6–15], while also rationalizing prescription patterns and enhancing operational efficiency in healthcare institutions [12,16–20]. However, comprehensive assessments of system-wide effects (e.g., per-visit costs, service utilization) remain limited, with existing studies predominantly regional in scope and yielding inconsistent conclusions. Some studies report significant reductions in total hospitalization costs and length of stay [14,21,22], whereas others highlight persistent growth in non-pharmaceutical expenditures and negligible long-term cost containment [23–25].

Following the nationwide implementation of VBP in 2019, the healthcare system faced compounded pressures from the COVID-19 pandemic and the parallel rollout of Diagnosis Related Group (DRG) payment reforms. This study employs an interrupted time series analysis (ITSA) with panel data to systematically evaluate the policy’s effects on per-visit costs, service utilization, and regional inequalities, providing empirical evidence for further policy optimization.

## Materials and methods

### Data sources

Healthcare related data, including per-visit outpatient (OP) expenditure, per-capita inpatient (IP) expenditure, the total number of hospital OP visits and admissions, and average length of stay were extracted from the *China Health Statistical Yearbook* (2010–2023) [26], an annual publication by National Health Commission of China. The data cover all public and private hospitals at primary, secondary, and tertiary levels in China and are aggregated provincial-level totals, not individual-level records. Consumer Price Index (CPI) and population data were obtained from the *China Statistical Yearbook* (2010–2023) [27], annually published by the National Bureau of Statistics of China. The regional analytical units are provincial-level jurisdictions in Mainland China, encompassing 22 provinces, 5 autonomous regions and 4 municipalities (referred to as 31 provincial units). China’s statistical yearbooks are published with a one-year lag; for example, the *2010 China Health Statistical Yearbook* contains the 2009 data. Consequently, all outcome variables therefore referred to the actual calendar years 2009–2022.

### Outcome variables

The primary outcomes included CPI-adjusted per-visit OP expenditure, per-capita IP expenditure, hospital OP rate, hospital IP rate, and average length of stay (LOS) from 2009 to 2022. The values were annual aggregates. Secondary outcomes assessed regional disparities in these indicators. All cost data were adjusted for inflation using annual CPI values and reported in Chinese Yuan (CNY).

- **Average cost per OP**: total outpatient revenue divided by the number of outpatient visits, unit: CNY per visit
- **Average cost per IP**: total inpatient revenue divided by the number of hospital discharges, unit: CNY per discharge
- **Hospital OP rate**: the total number of hospital OP visits divided by the population, unit: visit per person
- **Hospital IP rate**: the number of hospital admissions divided by the population, unit: percent (%)
- **Average length of stay**: total bed-days occupied by discharged patients divided by the number of discharges, unit: day

### Statistical analysis

The interrupted time-series (ITS) design collects outcome data at multiple time points before and after policy implementation. By controlling for pre-intervention trends in outcome variables, this approach enables statistical evaluation of policy effects without requiring parallel controls [28], making it widely applicable in public health and policy assessment. We conducted single group interrupted time series (ITS) analyses to evaluate the impact of the VBP policy.

The policy implementation year was set as 2019, with 2020 designated as the post-intervention period to account for policy lag. A segmented regression model was employed to quantify policy effects [29], expressed as:


$$Y_{t}=\beta_{\text{0}}+\beta_{\text{1}}\cdot Time+\beta_{\text{2}}\cdot Intervention+\beta_{\text{3}}\cdot Trend+\varepsilon_{t}$$

where:

- *Yₜ*: National-level outcome variable at time *t*, including health costs (average cost per OP and average cost per IP) and health utilization (hospital OP rates, hospital IP rate and average length of stay
- *Time*: Continuous variable measured in years, ranging from 2009 to 2022
- *Intervention*: Binary variable (0 = pre-implementation, 1 = post-implementation)
- *Trend*: Time elapsed since the intervention
- *β₂*: Immediate policy effect
- *β₃*: Sustained policy effect

Model selection followed the Akaike Information Criterion (AIC), with the minimal AIC model retained. Durbin–Watson test was performed to test the presence of first-order auto-correlation, a Durbin–Watson value obtained of around 2 indicates no sign of auto-correlation [30]. For datasets exhibiting autocorrelation, the Cochrane-Orcutt correction was applied to adjust model parameters [31]。

Regional heterogeneity analysis employed two-stage meta-analysis for year-region hierarchical data [32]. Stage 1 conducted province-specific ITS analyses, while Stage 2 assessed between-province heterogeneity using I² statistics. Effect sizes were pooled via fixed- or random-effects models based on heterogeneity tests, with results visualized using forest plots. R 4.4.3 was used for regression analysis. The significance level was set as two-sided α < 0.05.

## Results and discussion

### Impact of VBP policy on health costs

**Fig 1:**
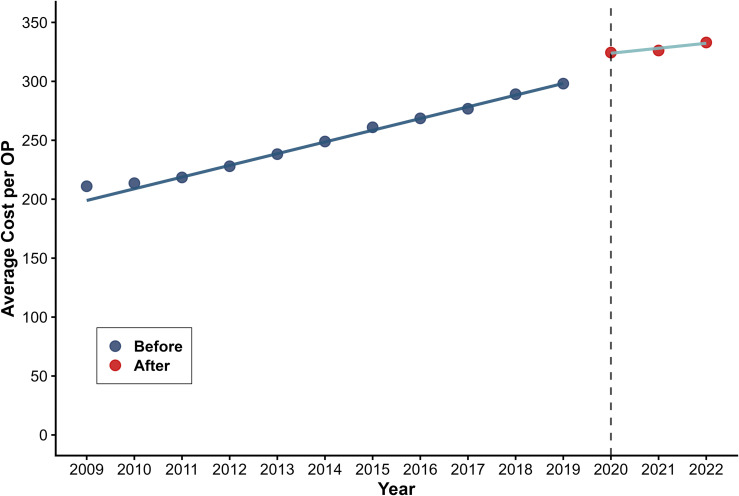
Trend in Average cost per OP.

**Fig 2:**
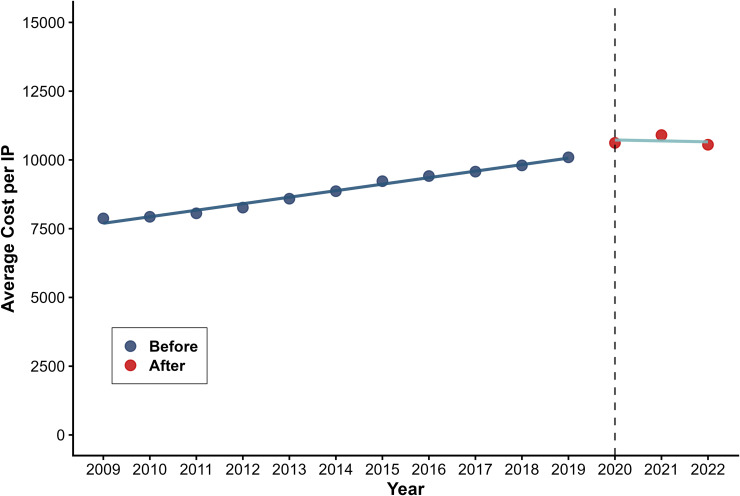
Trend in Average Cost per IP.

Table 1 presents the results of the segmented regression analysis used to predict two outcome variables for costs. The policy had significant effects on average costs per OP and IP. Average costs per OP increased instantaneously by CNY 21.400 (β_2_ = 21.400, P < 0.001) with a significant downward long-term trend (β_3_=−5.702, P < 0.001) (Fig 1). Average costs per IP increased by CNY 693.749 immediately post-intervention (β_2_ = 693.749, P = 0.006), followed by a progressive decline (β_3_=−270.670, P = 0.012) (Fig 2).

**Table 1:** Regression coefficients, standard errors and P-values from the segmented regression models predicting the outcomes for costs.

	coefficients	std. error	95% Confidence interval	P-value
**average costs per OP**	Cochrane-Orcutt adjusted			
*β*_*0*_	188.977	1.817	(184.866 − 193.087)	< 0.001
*β*_*1*_	9.930	0.243	(9.382 − 10.479)	< 0.001
*β*_*2*_	21.400	2.488	(15.771 − 27.030)	< 0.001
*β*_*3*_	−5.702	1.147	(−8.298 to −3.106)	< 0.001
**average costs per IP**				
*β*_*0*_	7459.466	79.577	(7282.158 − 7636.774)	< 0.001
*β*_*1*_	236.899	11.733	(210.756 − 263.042)	< 0.001
*β*_*2*_	693.749	200.378	(247.278 − 1140.220)	0.006
*β*_*3*_	−270.670	87.801	(−466.303 to −75.036)	0.012

### Impact of VBP policy on health utilization

**Fig 3:**
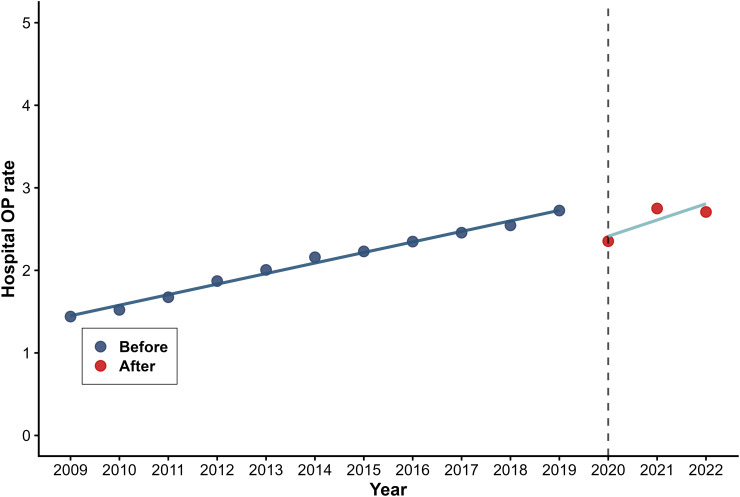
Trend in Hospital OP rate.

**Fig 4:**
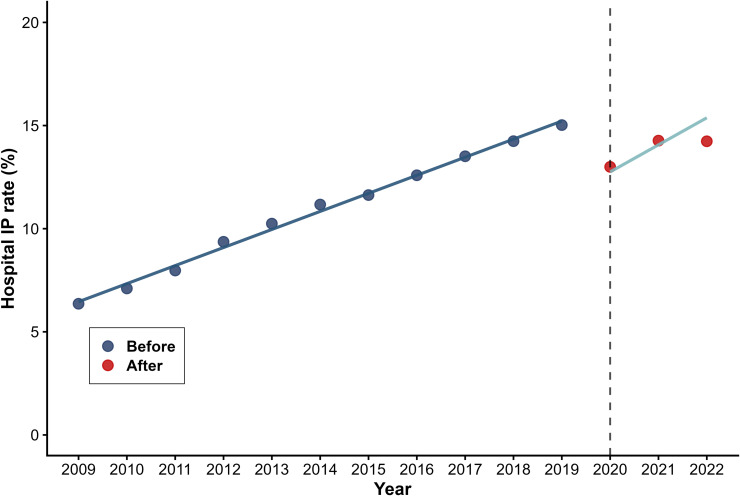
Trend in Hospital IP rate.

**Fig 5:**
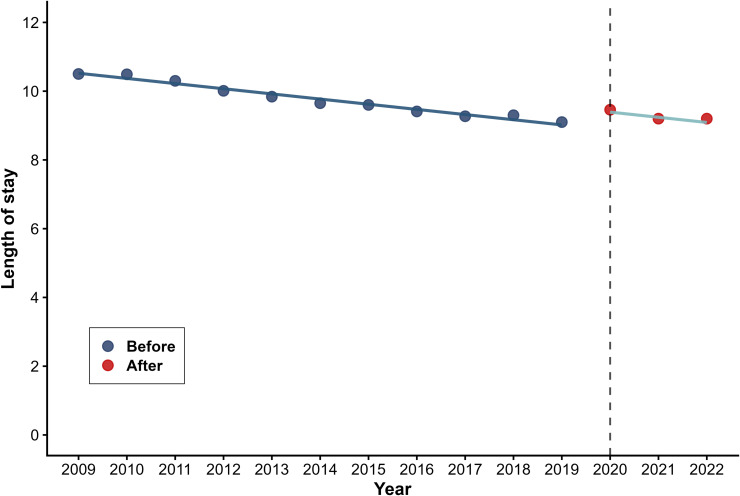
Trend in Length of stay.

Table 2 presents the results of the segmented regression analysis used to predict three outcome variables for health utilization volumes. Hospital OP rates decreased instantaneously by 0.510 visits per capita (β_2_=−0.510, P < 0.001) with a modest long-term upward trend emerged (β_3_ = 0.069, P = 0.046) (Fig 3). Hospital IP rate dropped abruptly by 3.775 percentage points (β_2_=−3.775, P < 0.001) (Fig 4), while average length of stay increased by 0.522 days immediately post-intervention (β_2_ = 0.522, P < 0.001) (Fig 5). No statistically significant long-term trends were observed for either hospital IP rate or length of stay.

**Table 2:** Regression coefficients, standard errors and P-values from the segmented regression models predicting the outcomes for volumes.

	coefficients	std. error	95% Confidence interval	P-value
**Hospital OP rates**				
*β*_*0*_	1.323	0.043	(1.227 − 1.418)	< 0.001
*β*_*1*_	0.128	0.006	(0.114 − 0.142)	< 0.001
*β*_*2*_	−0.510	0.090	(−0.709 to −0.312)	< 0.001
*β*_*3*_	0.069	0.031	(0.002 − 0.136)	0.046
**Hospital IP rate (%)**				
*β*_*0*_	5.583	0.297	(4.929 − 6.237)	< 0.001
*β*_*1*_	0.876	0.044	(0.779 − 0.972)	< 0.001
*β*_*2*_	−3.775	0.620	(−5.139 to −2.411)	< 0.001
**Average length of stay**				
*β*_*0*_	10.672	0.062	(10.537 − 10.806)	< 0.001
*β*_*1*_	−0.150	0.009	(−0.170 to −0.130)	< 0.001
*β*_*2*_	0.522	0.089	(0.329 − 0.715)	< 0.001

### Regional heterogeneity analysis

**Fig 6:**
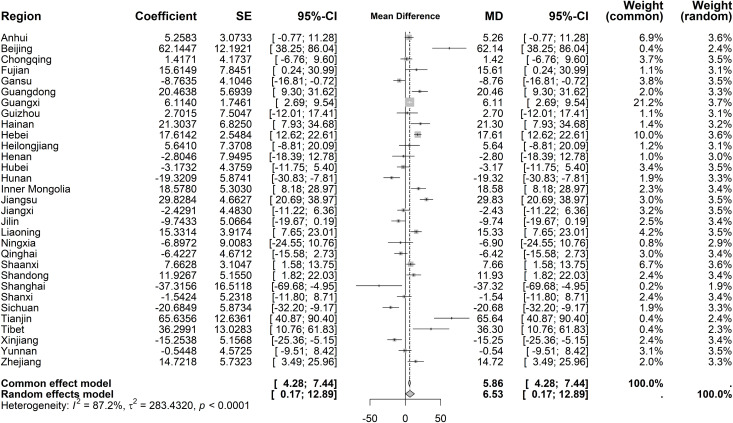
Forest Plot of Two-Stage ITS Analysis (intervention) for average costs per OP.

**Fig 7:**
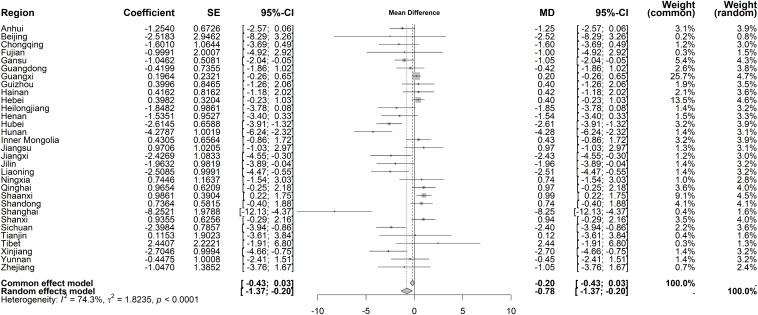
Forest Plot of Two-Stage ITS Analysis (trend) for average costs per OP.

For immediate policy effects on average costs per OP, substantial regional heterogeneity was observed (I² = 87.2%, *P* < 0.001), necessitating a random-effects model. The pooled immediate effect showed a marginal increase (mean difference [MD] = 6.53 CNY, 95% CI: 0.17–12.89) (Fig 6). Beijing and Tianjin exhibited the largest immediate increases (62.14 CNY and 65.64 CNY, respectively), while Shanghai had the most pronounced decrease (−37.32 CNY). For long-term trends, heterogeneity remained significant (I² = 74.3%, *P* < 0.001). Under the random-effects model, average costs per OP demonstrated a declining long-term trend (MD = −0.78 CNY, 95% CI: −1.37 to −0.20), with Shanghai showing the steepest reduction (−8.25 CNY) (Fig 7).

**Fig 8:**
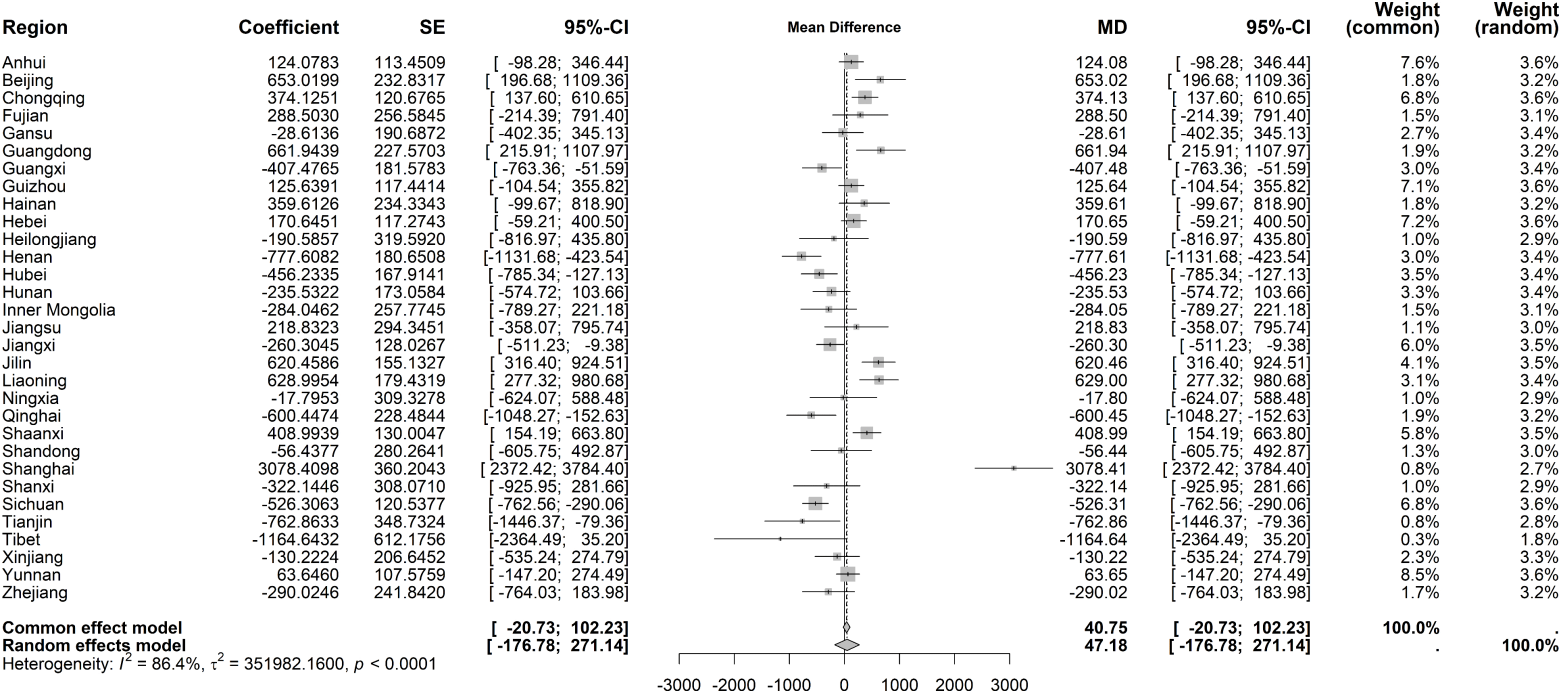
Forest Plot of Two-Stage ITS Analysis (intervention) for average costs per IP.

**Fig 9:**
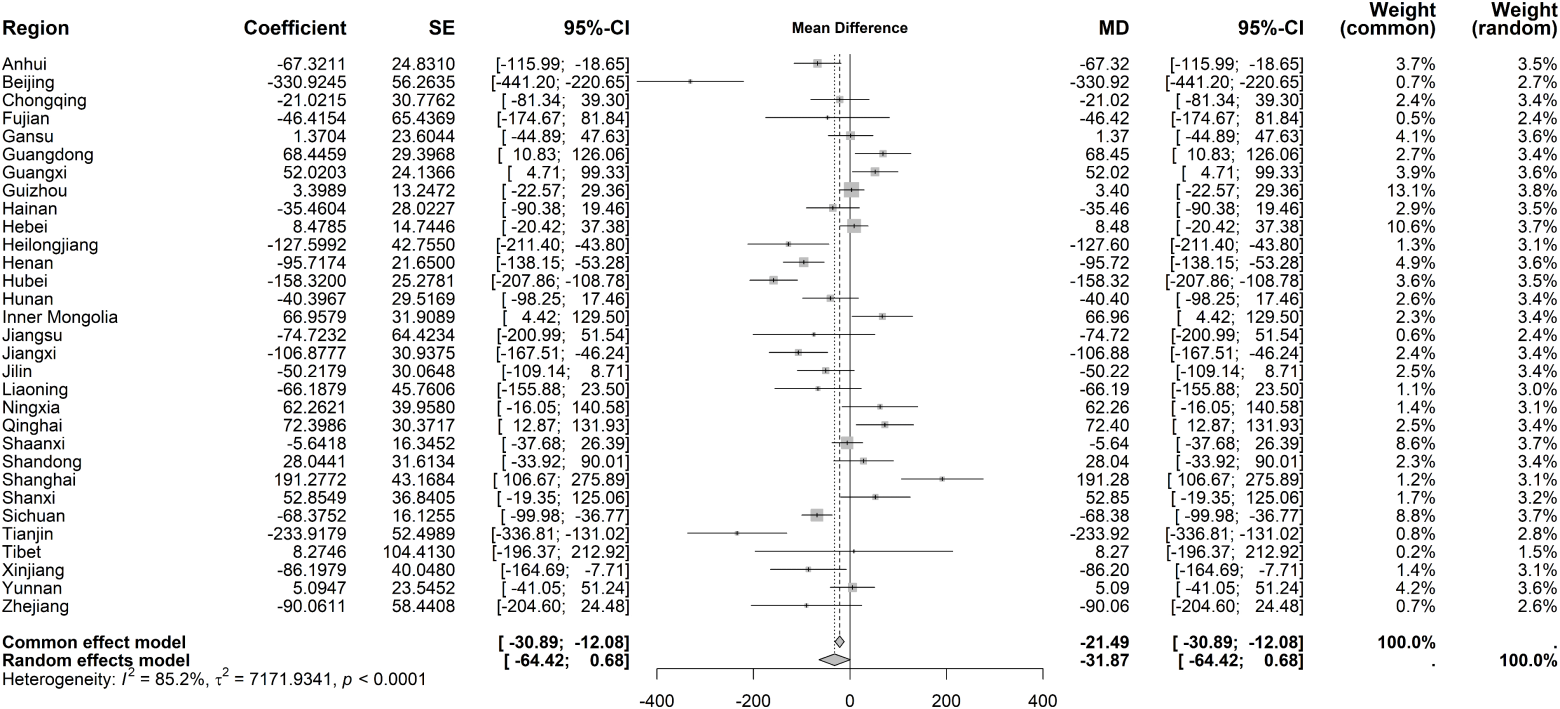
Forest Plot of Two-Stage ITS Analysis (trend) for average costs per IP.

High heterogeneity of average costs per IP was observed for immediate effects (I² = 86.4%, *P* < 0.001). The random-effects model revealed no statistically significant immediate change (MD = 47.18 CNY, 95% CI: −176.78 to 271.14), though Shanghai experienced a sharp rise (3078.41 CNY) (Fig 8). Long-term trends also displayed significant heterogeneity (I² = 85.2%, *P* < 0.001), with no overall significant trend under the random-effects model. However, Shanghai showed a marked long-term increase (191.28 CNY), while Beijing and Tianjin had significant declines (−330.92 CNY and −233.92 CNY, respectively) (Fig 9).

**Fig 10:**
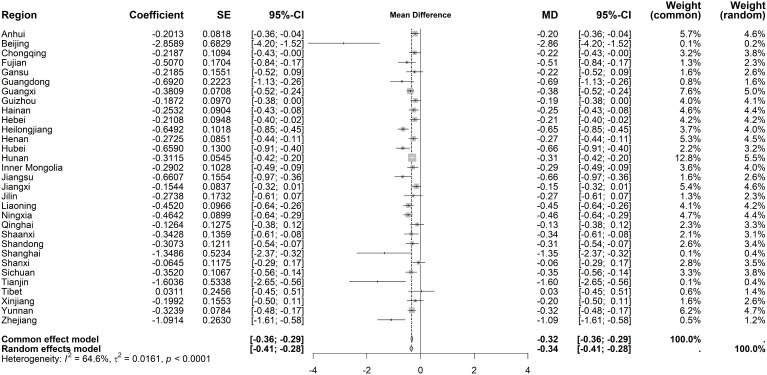
Forest Plot of Two-Stage ITS Analysis (intervention) for hospital OP rate.

**Fig 11:**
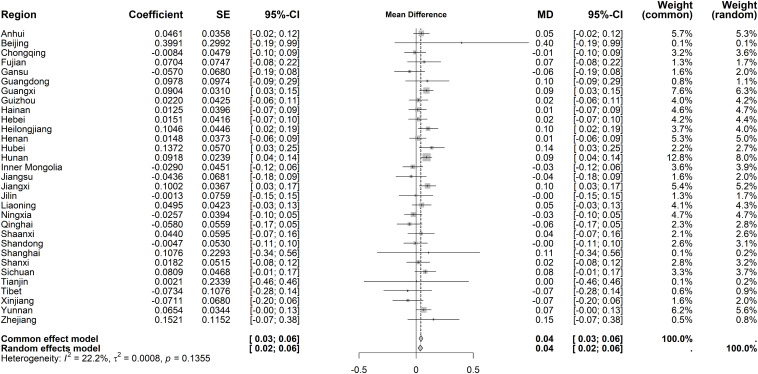
Forest Plot of Two-Stage ITS Analysis (trend) for hospital OP rate.

For immediate effects on hospital OP rates, heterogeneity was substantial (I² = 64.6%, *P* < 0.001). The random-effects model indicated a slight decrease (MD = −0.34 visits per capita, 95% CI: −0.41 to −0.28), with Beijing, Tianjin, Shanghai, and Zhejiang showing the largest reductions (>1 visit) (Fig 10). Long-term trends exhibited low heterogeneity (I² = 22.2%, *P* = 0.136), prompting a fixed-effects model. A marginal upward trend was observed (MD = 0.04 visits per capita, 95% CI: 0.03–0.06), though most regions showed no statistical significance (Fig 11).

**Fig 12:**
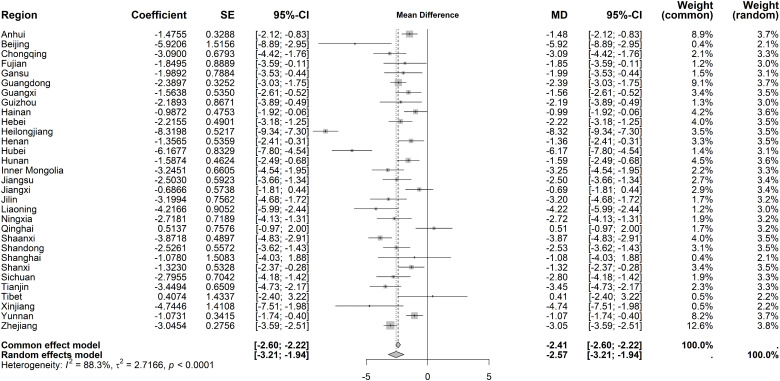
Forest Plot of Two-Stage ITS Analysis (intervention) for hospital IP rate.

**Fig 13:**
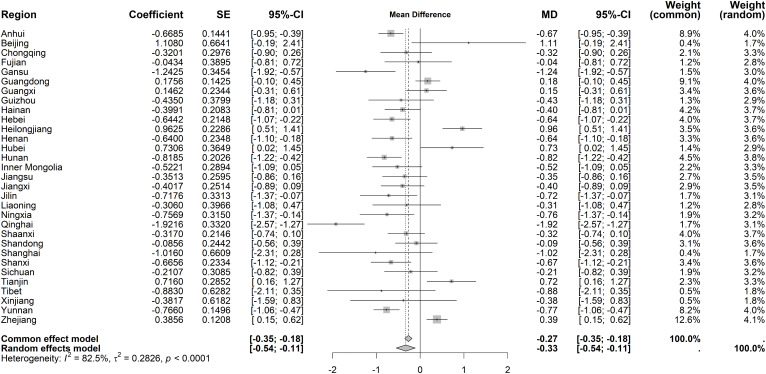
Forest Plot of Two-Stage ITS Analysis (trend) for hospital IP rate.

Immediate effects on hospital IP rate demonstrated high heterogeneity (I² = 88.3%, *P* < 0.001). The random-effects model revealed a significant decrease (MD = −2.57 percentage points, 95% CI: −3.21 to −1.94), with Heilongjiang and Hubei showing the largest declines (−8.32% and −6.17%, respectively) (Fig 12). Long-term trends also exhibited substantial heterogeneity (I² = 82.5%, *P* < 0.001), with a slight overall decline under the random-effects model (MD = −0.33 percentage points, 95% CI: −0.54 to −0.11). Notably, Heilongjiang, Hubei, and Tianjin displayed significant upward trends (0.96%, 0.73%, and 0.72%, respectively) (Fig 13).

**Fig 14:**
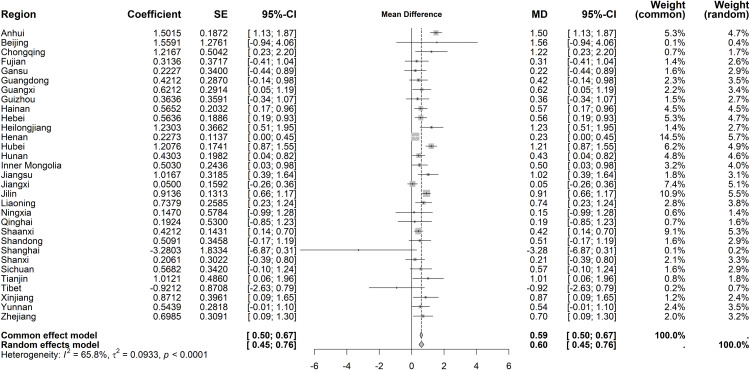
Forest Plot of Two-Stage ITS Analysis (intervention) for average length of stay.

**Fig 15:**
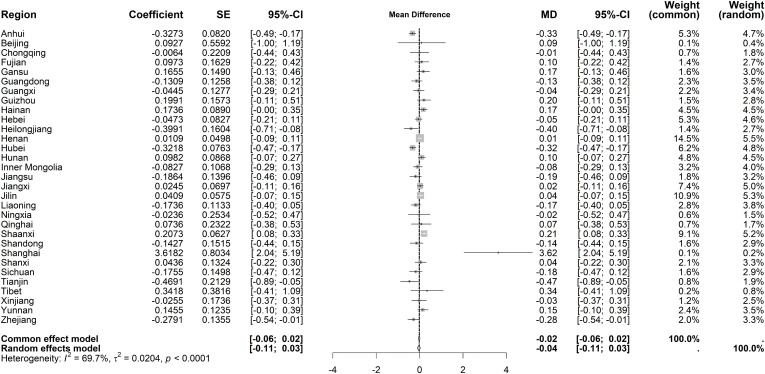
Forest Plot of Two-Stage ITS Analysis (trend) for average length of stay.

Immediate effects on average length of stay showed moderate heterogeneity (I² = 65.8%, *P* < 0.001). The random-effects model indicated a small increase (MD = 0.60 days, 95% CI: 0.45–0.76), though most regions lacked statistical significance (Fig 14). For long-term trends, heterogeneity persisted (I² = 69.7%, *P* < 0.001), but no significant overall trend emerged under the random-effects model (Fig 15).

### Theoretical implications

Since its implementation in 2019, China’s Volume-Based Procurement (VBP) policy has been accompanied by notable changes in healthcare utilization and costs. Our study identifies evolving short- and long-term patterns of these changes and contributes to health policy evaluation by integrating interrupted time series analysis (ITSA) with panel data to capture heterogeneous policy effects. These findings highlight the need for continued monitoring to further clarify the durability and magnitude of the observed effects. As the analysis is observational, the results should be interpreted as associational rather than causal.

The immediate post-policy increase in per-visit costs may reflect healthcare institutions’ revenue compensation strategies during the initial implementation phase. Studies have demonstrated that the VBP policy significantly reduced expenditures on targeted drugs, primarily driven by structural effects. However, overall spending remained uncontrolled due to increased utilization of substitute drugs and rising non-pharmaceutical costs [22,23,25]. Furthermore, irrational adjustments to healthcare cost structures may exacerbate patients’ financial burdens [24,33]. Over the long term, the VBP policy facilitated the release of drug price dividends. Post-implementation, a primary market-oriented drug pricing mechanism emerged [3,34], substantially improving medication affordability [7]. With increased utilization of procured drugs and sustained price reductions, this policy shows promise to further curb unreasonable growth in medical expenditures.

The COVID-19 pandemic in 2020 exerted unprecedented pressure on healthcare systems, leading to a sharp decline in service utilization. Data from China’s health statistics report indicated that total medical visits declined by approximately 10% year-on-year in 2020. A World Health Organization (WHO) survey revealed disruptions to 25 core healthcare services in 90% of countries, with non-communicable disease management being the second most affected [35]. Despite pandemic containment after April 2020, healthcare utilization recovered to only 80%–89% of pre-pandemic levels [36]. Resource diversion and stringent containment measures likely compromised healthcare accessibility, potentially confounding the evaluation of VBP effects.

The rebound in service utilization may stem from improved affordability due to reduced drug costs, which released pent-up patient demand. Simultaneously, providers may have engaged in demand-inducing practices [37]. Pandemic-related demand suppression and secondary health risks may have caused irreversible health capital depletion, further driving healthcare utilization [38,39]. Additionally, the sustained recovery in hospitalization rates could be attributed to synergies with Diagnosis-Related Group (DRG) payment reforms. DRG policies shorten average hospital stays and enhance bed turnover efficiency [40,41], indirectly expanding service capacity and driving utilization growth.

### Practical implications

Recently, DRG reforms and VBP policies have synergistically reduced costs through supply-demand co-regulation mechanisms, and their combined effects require further investigation. DRG incentivizes hospitals to optimize prescribing practices, prioritize procured drugs, and improve institutional efficiency [42]. Some regions have implemented innovative linkage mechanisms between DRG pricing benchmarks and VBP drug prices to amplify cost containment. However, policy overlap may induce distortions in medical practices, such as avoidance of high-risk procedures or delays in adopting innovative therapies, warranting vigilance toward unintended consequences. Decision-makers should account for overlapping policies to avoid underestimating budgetary impacts and to ensure coherent coordination of payment adjustments across different schemes.

Regional heterogeneity in policy effects reflects differences in local healthcare priorities, aging demographics, and behavioral adaptations among providers and patients. Regions with a higher degree of population ageing have experienced the most rigid increases in hospitalization costs [27]. Beijing initiated medical service price reforms in 2017, adopting a “one increase, two reductions” strategy: elevating fees for clinician labor-intensive services, reducing prices for advanced imaging, and eliminating drug markups through centralized procurement [43]. Concurrently, enhanced clinical pathway management further optimized care delivery, amplifying policy effectiveness. Provincial health commissions should pair VBP roll-outs with region-specific monitoring dashboards, allowing timely payment-rule adjustments before cost growth becomes entrenched.

## Limitations

Due to data availability, this study is constrained by the relatively short post-intervention observation window (2020–2022) and the reliance on annual data granularity, which may obscure quarterly fluctuations. While the data provides a valuable macro-level perspective, it does not account for heterogeneity in patient visit frequency or clinical severity. Future research should incorporate high-frequency and more granular data (e.g., quarterly or monthly data by clinical department) and extend the timeline to assess long-term sustainability. Additionally, qualitative insights into prescriber and patient decision-making could further enrich the interpretation of underlying mechanisms.

## Conclusions

The Chinese national centralized volume-based drug procurement policy appears to have lowered drug prices but was initially associated with increases in non-drug expenditures. Regional disparities linked to aging demographics and complementary reforms (e.g., DRG) may have influenced these outcomes, while the COVID-19 likely caused a temporary decline in utilization. Continued monitoring and the availability of longer-term data are required to validate these patterns and to inform the integration of DRG reforms with regionally tailored strategies that balance cost containment and service quality.

## Supporting information

S1 Table Health costs raw data. (XLSX)

S2 Table Health utilization raw data. (XLSX)

## Abbreviations

VBP: volume-based procurement
OP: outpatient
IP: inpatient
DRG: Diagnosis Related Group
ITSA: interrupted time series analysis
ITS: interrupted time series
CPI: Consumer Price Index
AIC: Akaike Information Criterion
MD: mean difference
WHO: World Health Organization
CNY: Chinese Yuan.
